# Polymorphisms Within DNA Double-Strand Breaks Repair-Related Genes Contribute to Structural Chromosome Abnormality in Recurrent Pregnancy Loss

**DOI:** 10.3389/fgene.2021.787718

**Published:** 2021-12-23

**Authors:** Zhenbo Cheng, Dehua Cheng, Jiancheng Li, Lihuang Guo, Wei Zhang, Conghui Zhang, Yangxu Liu, Yue Huang, Keqian Xu

**Affiliations:** ^1^ Department of Laboratory Medicine, The Third Xiangya Hospital, Central South University, Changsha, China; ^2^ Department of Laboratory Medicine, Xiangya School of Medicine, Central South University, Changsha, China; ^3^ School of Basic Medical Science, Institute of Reproductive and Stem Cell Engineering, Central South University, Changsha, China; ^4^ Reproductive and Genetic Hospital of CITIC-Xiangya, Changsha, China

**Keywords:** structural chromosome abnormalities, gene polymorphisms, DNA double-strand breaks, non-homologous end joining, EP300, whole-exome sequencing, recurrent pregnancy loss

## Abstract

**Background:** Structural chromosome abnormality (SCA) is an important cause of human diseases, including recurrent pregnancy loss (RPL). DNA double-strand breaks (DSBs) repair-related genes play critical roles in SCA. The present study aims to investigate the potential contribution of DSBs repair-related gene polymorphisms to SCA.

**Methods:** Fifty-four affected RPL individuals with SCA, 88 affected RPL individuals without SCA, and 84 controls were analyzed. Targeted whole-exome sequencing (WES) was used for screening single nucleotide polymorphisms in six DSBs repair-related genes (*EP300, XRCC6, LIG4, XRCC4, PRKDC*, and *DCLRE1C*), and validation was performed by Sanger sequencing. Finally, we detected the frequency of radiation-induced chromosome translocations in no SCA samples with significant polymorphisms by fluorescence *in situ* hybridization (FISH).

**Results:** A total of 35 polymorphisms have been identified and confirmed. Frequencies of *EP300* rs20551, *XRCC6* rs132788, and *LIG4* rs1805388 were significantly different between SCA RPL and no SCA RPL (*p* = 0.030, 0.031, and 0.040 respectively). Frequencies of those three gene polymorphisms between SCA RPL and controls also were significantly different (*p* = 0.017, 0.028, and 0.029 respectively). Moreover, the frequency of the G allele at rs20551 locus, the T allele at rs132788 locus and the A allele at rs1805388 locus was significantly higher in SCA RPL than no SCA RPL (*OR* = 3.227, *p* = 0.005; *OR* = 1.978, *p* = 0.008 and *OR* = 1.769, *p* = 0.036 respectively) and controls (*OR* = 7.130, *p* = 0.000; *OR* = 2.157, *p* = 0.004; *OR* = 2.397, *p* = 0.003 respectively). Additionally, the frequency of radiation-induced translocation in no SCA samples with rs20551, rs132788 or rs1805388 was significantly higher compared with the wild type samples (*p* = 0.015, 0.012, and 0.007 respectively).

**Conclusion:** Our results suggest that rs20551, rs132788, and rs1805388 might be associated with the risk of SCA. Larger scales of genetic variations studies and functional experiments are necessary to further confirm these findings.

## Introduction

Structural chromosome abnormality (SCA) is an important cause of human diseases including recurrent pregnancy loss (RPL) (Rai and Regan, 2006). In approximately 2–5% of couples with RPL, one partner (more often the woman) will have a genetically balanced SCA (RCOOG, 2011).

Types of SCA include translocation, inversion, deletion, Tandem duplication, ring chromosome, etc. (Morin et al., 2017; Menghi et al., 2018; Panday et al., 2021). The most common SCA in women with RPL is translocation (usually 60% reciprocal and 40% Robertsonian approximately), and the segregation during meiosis can result in gametes with duplication or deficiency of chromosome segments (Prosée et al., 2020). Chromosome inversion is also associated with a higher risk of RPL, and the risk of RPL is affected by the size and genetic content of the rearranged chromosomal segments (Nagirnaja et al., 2014; Page and Silver, 2016).

The biogenesis of SCA is remarkably poorly understood. Generally, the formation of SCA is considered a multistep process, and the initial event is the concomitant occurrence of DNA double-strand breaks (DSB) in multiple chromosomal locations (Nambiar and Raghavan, 2011). It is generally agreed that DSBs repair, especially non-homologous end joining (NHEJ) repair, plays an important role in the formation of SCA (Chang et al., 2017).

The human *EP300*, *XRCC6*, *LIG4*, XRCC4, *PRKDC*, and *DCLRE1C* were identified as playing critical roles in NHEJ repair (Tropberger et al., 2013; Wang et al., 2013; Ochi et al., 2015; Manickavinayaham et al., 2019). *EP300* encodes the E1A binding protein p300 which functions as histone acetyltransferase and regulates transcription *via* chromatin remodeling (Tropberger et al., 2013). *XRCC6* locates on chromosome 22q13, coding the X-ray repair cross-complementing protein 6 (also named Ku70), which can be readily participated in repairing a DSB (Zhao et al., 2020). Moreover, DNA LIG4 is also essential for DSBs repair (Grawunder et al., 1998). The protein encoded by *XRCC4* functions together with DNA LIG4 and the DNA-dependent protein kinase in the repair of DSBs (Zolner et al., 2011), and polymorphisms within these genes have been shown contributing to cancers and other disorders caused by genomic instability (Singh et al., 2018; Garcia et al., 2019). *PRKDC* encodes the catalytic subunit of DNA-dependent protein kinase (DNA-PKcs), is a candidate regulator of DSBs repair (Bunting and Nussenzweig, 2013). Additionally, *DCLRE1C* encodes Artemis, as one co-chaperone of DNA-PKcs, could bind to Ku70-Ku80-DNA complex and processes the DSBs (Bunting and Nussenzweig, 2013). We hypothesize that polymorphisms within those six DSBs repair related genes might contribute to the formation of SCA.

In the present study, we investigated the potential contribution of *EP300*, *XRCC6*, *LIG4*, XRCC4, *PRKDC*, and *DCLRE1C* gene polymorphisms to structural chromosome abnormalities (SCA) based on recurrent pregnancy loss. We used targeted WES in a relatively small exploratory sample at the first stage, and then confirmed by Sanger sequencing in a lager cohort including all exploratory sample and confirmatory sample. Finally, we also detected the frequency of radiation-induced chromosome translocations in no SCA samples with significant polymorphisms by fluorescence *in situ* hybridization (FISH).

## Materials and Methods

### Ethics Approval Statement

The study was approved by the Ethics Committee of the Third Xiangya Hospital (Quick 19159). Informed consent was obtained from all subjects involved in the study.

### Study Subjects

The 142 affected individuals, all were RPL (54 with SCA and 88 without SCA), had no history of endocrine, metabolic, autoimmune, or other systemic disorders, thrombophilia, or uterine anatomic abnormalities. We recruited the RPL in strict accordance with the Practice Committee of the American Society for Reproductive Medicine (Practice Committee of the American Society for Reproductive Medicine, 2020). The controls included 84 age-matched fertile women in pregnancy and had no history of complicated pregnancies, miscarriages, still births, small for gestational age fetuses, preeclampsia, ectopic pregnancy, preterm delivery or any other pregnancy complication. Chromosomal abnormalities were excluded in the control by karyotype results. The demographic and clinical characteristics also were collected.

**FIGURE 1:**
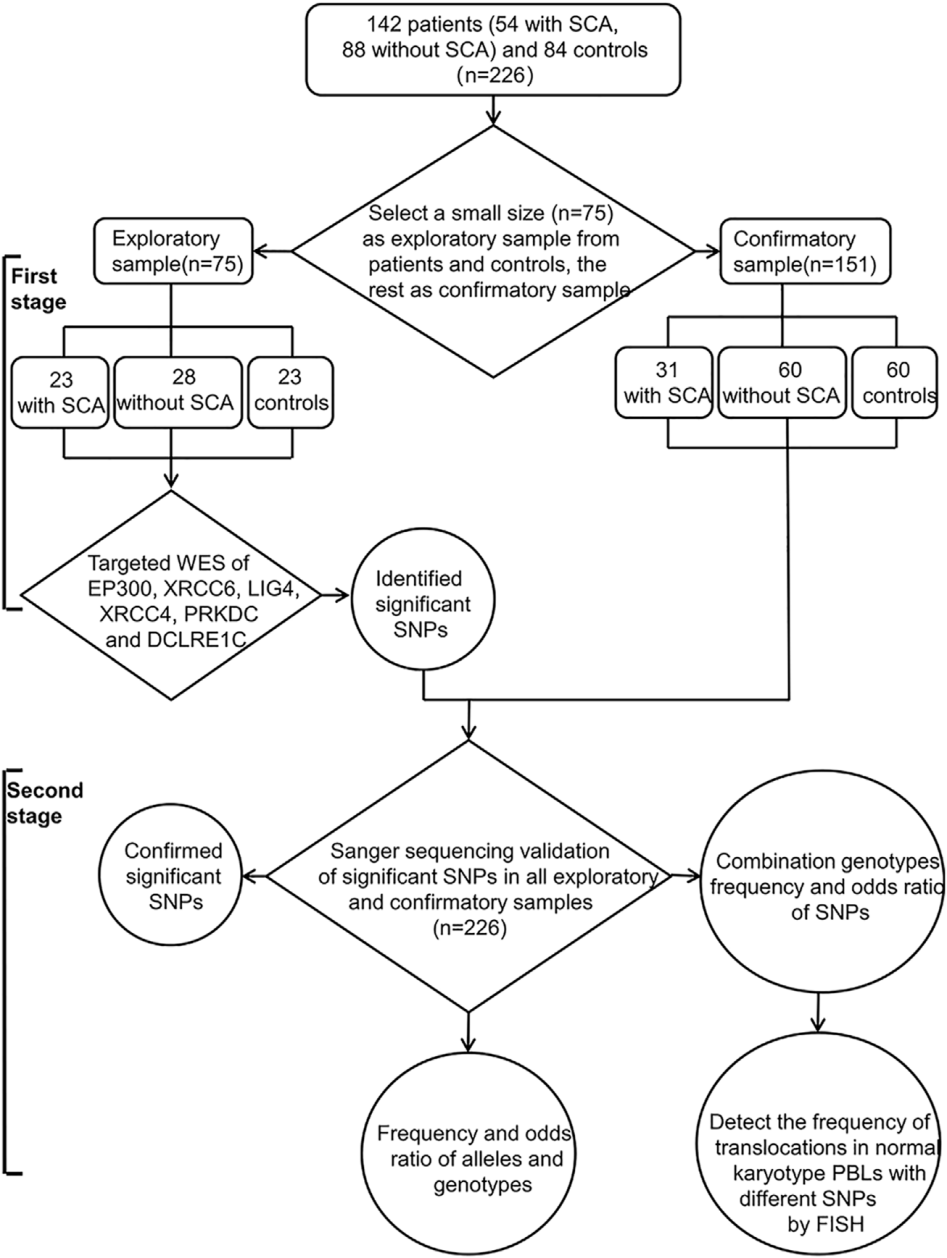
Flowchart of this study design. There are two stages in our study: The first stage, to identify the significant SNPs by targeted WES in a relatively small size exploratory sample (*n* = 75); Second, validation in a larger sample size (*n* = 226, exploratory and confirmatory sample) using Sanger sequencing, and then detect the frequency of radiation-induced translocations in normal karyotype PBLs with different genotype by FISH. SCA: Structural chromosome abnormalities; WES: Whole-exome sequencing; SNPs: Single nucleotide polymorphisms; PBLs: Peripheral blood lymphocytes; FISH: fluorescent *in situ* hybridization.

The flowchart for the study design was shown in Figure 1. We first used targeted WES to identify significant SNPs in relatively small exploratory samples (*n* = 75) at the first stage, and then confirmed by Sanger sequencing in a larger cohort (*n* = 226) including all exploratory samples (*n* = 75) and confirmatory samples (*n* = 151). Finally, to further confirm the association of significant SNPs with SCA, we detected the frequency of radiation-induced (2Gy X-ray) chromosome translocations in normal karyotype RPL peripheral blood lymphocytes (PBLs) with significant gene polymorphisms by FISH.

### Peripheral Blood Karyotype Analysis

A standard 72-h lymphocyte culture of peripheral blood (2–5 ml) from each patient was performed to produce Metaphases for karyotyping. G banding was performed by a trypsin pretreatment of chromosomes followed by Giemsa staining. Chromosomes’ analysis was done using MetaSystems Ikaros (ZEISS, Germany) and karyotypes were reported according to International System for Human Cytogenetic Nomenclature (Simons et al., 2013). Karyotype analysis was performed using at least 20 Metaphases for each sample. The number was expanded to 100 metaphases in case of suspected mosaicism.

### Screening Single Nucleotide Polymorphisms by Targeted Whole-Exome Sequencing

We first detected 75 samples (23 with SCA, 28 without SCA and 24 controls) by targeted whole-exome sequencing (WES). Genotyping of SNPs was performed with the WES-based targeted sequence analysis and Sanger sequencing. The library was constructed with the kit (Vazyme VAHTS UniveRPLl Plus DNA Library Prep Kit for Illumina, United States) by the standard procedure according to the manufacturer’s instructions. xGen® Lockdown® Probes (Nanodigmbio, United States, Sequences presented in Supplementary Table S1) were used to capture the target genes. Sequencing was carried out in NovaSeq 6000 (Illumina). FastQC was used to filter the raw data. The sequenced reads were aligned to the human reference genome 19 (HG19) using BWA MEN, and PCR duplicates were marked with PICARD. Variants were called by GATK HaplotypeCaller with default parameters, and retained considering DP (reads depth) ≥8, MQ (Mapping Quality) ≥20. Variation annotation was done in ANNOVAR software, variants with VAF≤0.01 [1000 genome project (2015) and ExAC Project] in the coding region and splicing site were filtered out, and the VAF >0.01 were kept.

### SNPs Validation (Sanger Sequencing)

All significant SNPs detected were verified by Sanger sequencing (ABI 3730XL, United States). SNPs were reported according to Human Genome Variation Society nomenclature (Dunnen and Antonarakis, 2000). The sequences for PCR primers are listed in Supplementary Table S2.

### Detection of the Translocations by Fluorescence *in situ* Hybridization (FISH)

FISH was used to detect the radiation-induced chromosome translocations in peripheral blood lymphocytes (PBLs) from normal karyotype RPL after 2Gy X-rays as previously described (Nakano et al., 2001). Metaphases were harvested after co-cultured with colchicine for 2 h. Chromosomes 1 and 4 were painted green by *in situ* hybridization with composite probes labeled with SYBR green (Cytocell, United Kingdom), chromosomes 2 were painted red by *in situ* hybridization with composite probes labeled with Rhodamine B (Cytocell, United Kingdom). The observed frequency of translocations (*F*_p_) detected by FISH represents the frequency between painted chromosomes 1, 2, and 4 and the remaining counterstained chromosomes. To compare *F*_p_ with the values for translocations detected by the conventional method that detects aberrations involving the entire chromosome set, it is necessary to estimate the genome-equivalent frequency of translocations (*F*_G_). Thus, since the fraction of the total genomic DNA content represented by painted chromosomes 1, 2, and 4 to the total genome is 0.228 for males and 0.224 for females, *F*_p_ was multiplied by 2.771 for males and 2.806 for females to estimate *F*_G_; the basic method used is essentially that described by Pearce (Pearce et al., 2012). 400 metaphase splitting images were observed for each sample by three observers. The experiments were repeated three times.

### Statistical Analysis

All statistical analyses were performed using SSPS 25.0 (IBM Corp, Chicago). Prism 7.0 software (GraphPad, United States) was utilized to draw figures. Qualitative data were expressed as *n* (%) and analyzed using Pearson chi-square (χ^2^) test (*n* ≥ 40 and expected frequencies ≥5). Measurement data were expressed as‾x (SD). Differences in genotype and allele frequencies (*n* < 40 or expected frequencies <5) between affected individuals and controls, as well as deviations from Hardy-Weinberg equilibrium were determined using Fisher exact test. Odds ratios (ORs) and 95% confidence intervals (CIs) were calculated to evaluate the contribution of *EP300*, *XRCC6*, *LIG4*, *XRCC4*, *PRKDC*, and *DCLRE1C* gene polymorphisms to SCA. *p* < 0.05 was considered to be statistically significant.

## Results

### Demographic and Clinical Characteristics of Subjects

The demographic and clinical characteristics of the affected individuals and controls are summarized in Table 1. Overall, the mean age, education level, body mass index, smoking, alcohol use, menarche age, menstrual cycle, age of pregnancy, TSH, PRL, Ureaplasma urealyticum, *Mycoplasma* hominus, Toxoplasma gondii, rubella, cytomegalovirus, herpes virus, LA, ß2-GPI, and aCL were similar between affected individuals and controls. No significant differences were identified (*p* > 0.05) (Table 1).

**TABLE 1:** Demographic and clinical characteristics of the affected RPL individuals and the controls. SD = standard deviation.

Characteristic	Variable	RPL with SCA	Control	*p*	RPL with SCA	RPL without SCA	*p*	RPL without SCA	Control	*p*
		(*n*=54)	(*n*=84)		(*n*=54)	(*n*=88)		(*n*=88)	(*n*=84)	
Age, years, mean (SD)	—	32.3 (4.2)	28.8(6.4)	0.487	32.3 (4.2)	31.4 (4.7)	0.847	31.4 (4.7)	28.8 (6.4)	0.805
Education, *n* (%)	Primary Diploma	3 (5.6)	1 (1.2)	0.313	3(5.6)	2 (2.3)	0.275	2 (2.3)	1 (1.2)	0.291
	Secondary Diploma	24 (44.4)	35 (41.7)	—	24(44.4)	46 (52.3)	—	46 (52.3)	35 (41.7)	—
	College Diploma	27 (50.0)	48 (57.1)	—	27(50.0)	40 (45.4)	—	40 (45.4)	48 (57.1)	—
BMI, kg/m^2^, mean (SD)	—	22.7(4.9)	23.5 (3.1)	0.295	22.7(4.9)	23.3 (3.4)	0.611	23.3 (3.4)	23.5 (3.1)	0.825
Smoking, *n* (%)	—	6(11.1)	11 (13.1)	0.675	6 (11.1)	12 (13.6)	0.473	12 (13.6)	11 (13.1)	0.746
Alcohol use, *n* (%)	—	15(27.8)	18 (21.4)	0.369	15(27.8)	19 (21.6)	0.375	19 (21.6)	18 (21.4)	0.799
Menarche age, years,mean (SD)	—	13.8 (1.2)	14.3 (1.5)	0.357	13.8 (1.2 )	13.2 (1.1)	0.804	13.2(1.1)	14.3 (1.5)	0.475
Menstrual cycle, days, mean (SD)	—	29.2 (4.7)	29.3 (3.1)	0.517	29.2(4.7)	29.5 (3.3)	0.615	29.5 (3.3)	29.3 (3.1)	0.715
Age of pregnancy, years, mean (SD)	—	28.2 (1.8)	27.3 (2.3)	0.295	28.2 (1.8)	27.3 (2.3)	0.701	27.3 (2.3)	27.2 (2.2)	0.515
Gestational age at loss, week, mean (SD)	—	—	—	—	8.9(2.7)	9.3 (2.4)	0.785	—	—	—
TSH, µIU/mL, mean (SD)	—	2.38 (0.71)	3.13 (1.10)	0.117	2.38 (0.71)	2.87 (0.97)	0.358	2.87 (0.97)	3.13 (1.10)	0.212
PRL, ng/mL, mean (SD)	—	36.73 (12.02)	47.73(17.02)	0.125	36.73(12.02)	38.69(11.82)	0.234	38.69(11.82)	47.73(17.02)	0.099
UU or Mh or CT, *n* (%)	—	8 (14.9)	11 (13.1)	0.082	8(14.9)	13 (14.8)	1.000	13 (14.8)	11 (13.1)	0.101
TORCH IgG or IgM, *n* (%)	—	15 (47.8)	18(33.3)	0.728	15(47.8)	22 (42.8)	0.134	22 (42.8)	18(33.3)	0.307
LA IgG or IgM or IgA, *n* (%)	—	3 (5.6)	6 (7.1)	0.136	3 (5.6)	6 (6.8)	0.764	6(6.8)	6 (7.1)	0.933
ß2-GPI, *n* (%)	—	3 (5.6)	5 (6.0)	0.924	3(5.6)	7 (8.0)	0.588	7 (3.6)	5 (6.0)	0.607
aCL, *n* (%)	—	1 (1.9)	0 (0.0)	—	1 (1.9)	1 (1.1)	0.726	1 (1.1)	0 (0.0)	—
Sonohysterography, *n* (%)	—	2 (3.8)	1 (1.2)	0.341	2 (3.8)	1 (1.1)	0.302	1(1.1)	1(1.2)	0.975

*Note:* The omission of *p*-value in the table was intentional because the number of cases was zero. SD = standard deviation; RPL Recurrent Pregnancy Loss; BMI = body mass index; TSH thyroid-stimulating hormone; PRL prolactin; UU Ureaplasma urealyticum; Mh = *Mycoplasma* hominus; TORCH Toxoplasma gondii, rubella, cytomegalovirus, herpes virus; LA Lupus anticoagulant; *ß*2-GPI *ß*2-glycoprotein I; aCL anticardiolipin antibody; SCA structural chromosome abnormalities.

### Distribution of Structural Chromosome Abnormalities (SCA)

There were **54** RPL with SCA: **32** carried a balanced reciprocal translocation, among them, chromosome 2 were involved in the translocation most frequently (*n* = 7) followed by chromosomes 1, 4, 11, and 12 (*n* = 5, each), chromosomes 3, 5, and 9 (*n* = 2, each), chromosomes 6, 17, and 19 were not involved; **8** carriers of inversions were observed, 7 were inversions of chromosome 9 and 1 inversion of chromosome 8; **14** carried a balanced Robertsonian translocation (Supplementary Table S3).

### Results of Sequencing

A total of 35 polymorphisms had been identified in our samples (Table 2), nine within *EP300*, two within *XRCC6*, four within *LIG4*, three within *XRCC4*, ten within *PRKDC* and seven within *DCLRE1C* by WES. In *EP300* polymorphisms, three were non-synonymous variants, six were synonymous variants. All *XRCC6* polymorphisms identified were synonymous variants, while all *LIG4* polymorphisms were non-synonymous variants and only one non-synonymous variant was identified in *XRCC4.* Additionally, most polymorphisms in *PRKDC* and *DCLRE1C* were non-synonymous variants (Table 2)*.* There was no missing data. The alleles and genotype frequencies of all the polymorphism loci in control were consistent with the Hardy-Weinberg equilibrium (*p* > 0.05, data not shown).

**TABLE 2:** Frequency of SNPs found in exploratory sample among affected RPL individuals and controls identified by the WES, *n* (%).

Gene SNPVarianttype	SCA RPL (*n* = 23)	Control (*n* = 24)	*χ* ^2^	*p*	SCA RPL (*n* = 23)	no SCARPL (*n* = 28)	Control (*n* = 24)	*χ* ^2^	*p*	no SCARPL (*n* = 28)	Control (*n* = 24)	*χ* ^2^	*p*
*EP300*													
rs20551	**N**	**7(30.4)**	**1(4.2)**	**5.738**	**0.017**	**7(30.4)**	**2(7.1)**	**4.714**	**0.030**	2(7.1)	1(4.2)	0.211	0.646
rs20552	S	23(100)	23(82.1)	0.979	0.322	23(100)	27(96.4)	0.838	0.360	27(96.4)	23(82.1)	0.012	0.913
rs20554	S	13(56.5)	13(46.4)	0.026	0.872	13(56.5)	10(35.7)	2.208	0.137	10(35.7)	13(46.4)	1.784	0.182
rs78045947	S	0(0)	1(4.2)	—	—	0(0)	2(7.1)			2(7.1)	1(4.2)	0.211	0.646
rs139551099	S	0(0)	1(4.2)	—	—	0(0)	2(7.1)			2(7.1)	1(4.2)	0.211	0.646
rs188035979	N	1(4.4)	0(0)	—	—	1(4.4)	1(3.6)	0.012	0.913	1(3.6)	0(0)	—	—
rs17002307	S	0(0)	0(0)	—	—	0(0)	1(3.6)			1(3.6)	0(0)	—	—
rs146242251	N	1(4.4)	1(4.2)	0	1.000	1(4.4)	1(3.6)	0.012	0.913	1(3.6)	1(4.2)	0.012	0.913
rs7575206	S	0(0)	0(0)	—	—	0(0)	1(3.6)	—	—	1(3.6)	0(0)	—	—
*XRCC6*													
rs132788	**S**	**16(69.5)**	**9(37.5)**	**4.850**	**0.028**	**16(69.5)**	**11(39.3)**	**4.647**	**0.031**	11(39.3)	9(37.5)	0.017	0.896
rs550596546	S	0(0)	0(0)	—	—	0(0)	1(3.6)	—	—	1(3.6)	0(0)	—	—
*LIG4*													
rs1805388	**N**	**14(60.9)**	**7(29.2)**	**4.776**	**0.029**	**14(60.9)**	**9(32.1)**	**4.209**	**0.040**	9(45.0)	7(36.8)	0.054	0.816
rs1805389	N	5(21.7)	3(12.5)	0.507	0.476	5(21.7)	5(17.9)	0.121	0.728	5(17.9)	3(12.5)	0.285	0.593
rs139713386	N	0	0	—	—	0	1(3.6)	—	—	1(3.6)	0	—	—
rs2232641	N	0	1(4.2)	—	—	0	0	—	—	0	1(4.2)	—	—
*XRCC4*													
rs1805377	A	20(87.0)	22(91.7)	0.274	0.600	20(87.0)	26(92.9)	0.497	0.481	26(92.9)	22(91.7)	0.026	0.872
rs3734091	N	7(30.4)	6(25)	0.173	0.677	7(30.4)	5(17.9)	1.110	0.292	5(17.9)	6(25)	0.395	0.530
rs1056503	S	20(87.0)	22(91.7)	0.274	0.600	20(87.0)	26(92.9)	0.497	0.481	26(92.9)	22(91.7)	0.026	0.872
*PRKDC*													
rs11411516	F	20(87.0)	19(79.2)	0.505	0.477	20(87.0)	20(71.4)	1.800	0.179	20(71.4)	19(79.2)	0.413	0.520
rs55769154	N	2(8.7)	1(4.2)	0.403	0.526	2(8.7)	1(3.6)	0.599	0.439	1(3.6)	1(4.2)	0.012	0.913
rs55793951	N	0	0	—	—	0	2(10.0)	—	—	2(10.0)	0	—	—
rs750714859	N	0	1(4.2)	—	—	0	0	—	—	0	1(4.2)	—	—
rs756127946	N	0	1(4.2)	—	—	0	0	—	—	0	1(4.2)	—	—
rs77033659	N	1(4.3)	0	—	—	1(4.3)	0	—	—	0	0	—	—
rs749856389	N	1(4.3)	0	—	—	1(4.3)	0	—	—	0	0	—	—
rs187813872	S	1(4.3)	0	—	—	1(4.3)	0	—	—	0	0	—	—
rs369274149	N	0	0	—	—	0	1(3.6)	—	—	1(3.6)	0	—	—
rs547031184	S	0	0	—	—	0	1(3.6)	—	—	1(3.6)	0	—	—
*DCLRE1C*													
rs7076862	S	13(56.5)	13(54.2)	0.051	0.821	13(56.5)	14(50.0)	0.114	0.736	14(50.0)	13(56.5)	0.011	0.916
rs35441642	N	6(26.1)	10(41.7)	2.063	0.151	6(26.1)	9(32.1)	0.960	0.327	9(32.1)	10(41.7)	0.633	0.227
rs12768894	N	7(30.4)	8(33.3)	0.208	0.648	7(30.4)	6(21.4)	0.539	0.463	6(21.4)	8(33.3)	0.931	0.335
rs183622528	N	0	1(4.2)	—	—	0	0	—	—	0	1(4.2)	—	—
rs7830743	S	5(21.7)	3(12.5)	0.507	0.476	5(21.7)	2(7.1)	2.272	0.132	2(7.1)	3(12.5)	1.232	0.267
rs8178235	S	2(8.7)	1(4.2)	—	—	2(10.0)	0	—	—	0	1(4.2)	—	—
rs8178245	N	0	2(10.4)	—	—	0	0	—	—	0	2(10.4)	—	—

Note: The omission of χ^2^ and *p*-value in the table was intentional because the number of cases was zero. Values in bold indicate statistically significantly (*p* < 0.05). SNPs: single nucleotide polymorphisms; WES whole-exome sequencing; RPL Recurrent Pregnancy Loss; SCA structural chromosome abnormalities; N = non-synonymous Variant; S = synonymous Variant; A = Splice Acceptor Variant; F = frameshift insertion.

**FIGURE 2:**
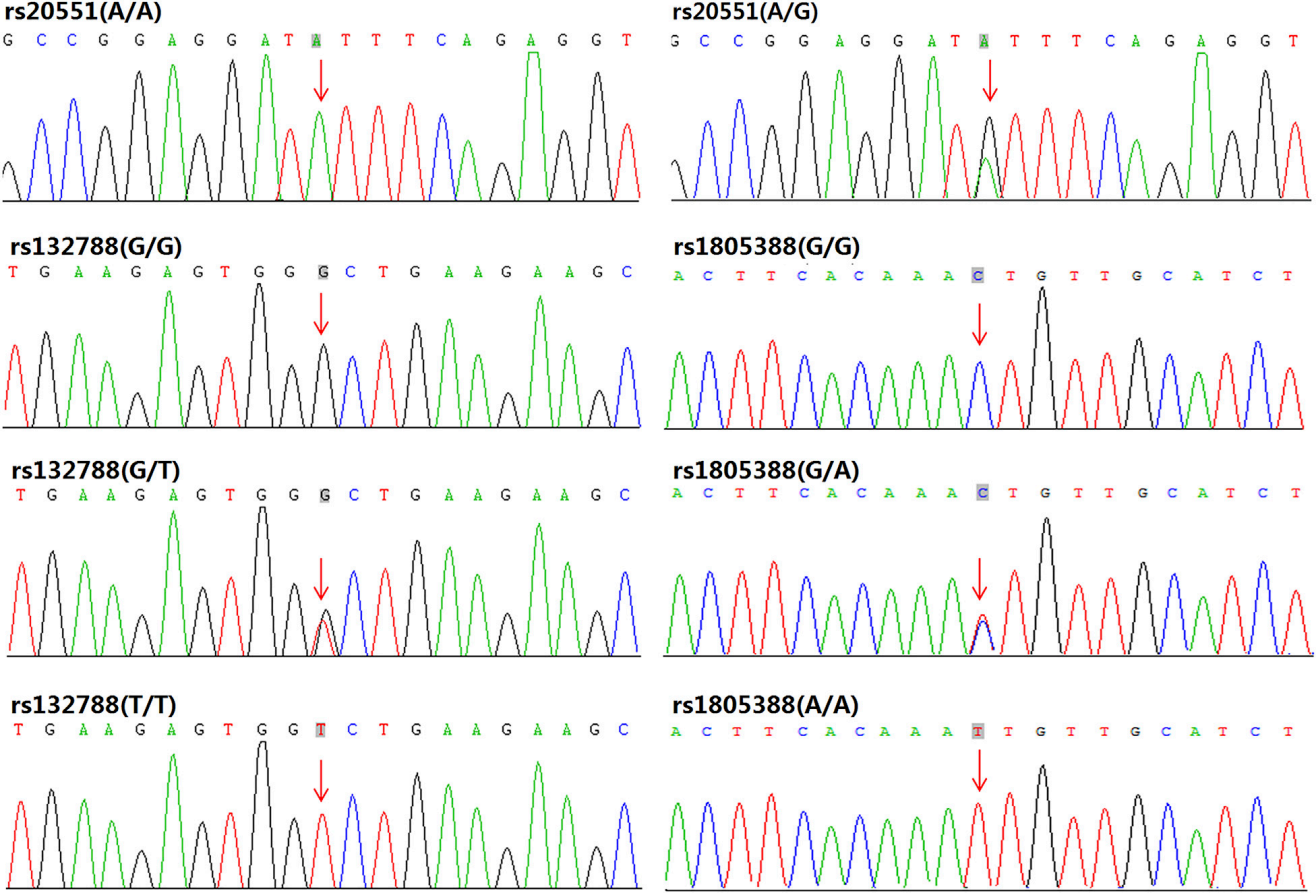
Sanger sequencing validation results for *EP300* rs20551, *XRCC6* rs132788, and *LIG4* rs1805388: In our samples, all the rs20551 loci were heterozygous of A/G (except for the wild type A/A), while rs132788 consisted of heterozygotes (G/T) and homozygotes (T/T), and rs1805388 consisted of heterozygotes (G/A) and homozygotes (A/A), the consistency with results from WES was 100%.

Frequencies of *EP300* rs20551, *XRCC6* rs132788, and *LIG4* rs1805388 were statistically significantly different between RPL with SCA and RPL without SCA group (*p* = 0.030, 0.031, 0.040 respectively). Frequencies of those three gene polymorphisms between RPL with SCA group and controls were also shown significantly different (*p* = 0.017, 0.028, and 0.029 respectively). All rs20551 were heterozygous, while rs132788 and rs1805388 consisted of heterozygotes and homozygotes, and verified by Sanger sequencing (Figure 2), the concordance rate was 100%. The frequency of the G allele at rs20551 locus, the T allele at rs132788 locus and the A allele at rs1805388 locus in SCA RPL was statistically significantly higher than the no SCA RPL (*OR =* 3.227, *p =* 0.005; *OR =* 1.978, *p =* 0.008; *OR =* 1.769, *p =* 0.036 respectively) and the control group (*OR* = 7.130, *p* = 0.000; *OR* = 2.157, *p* = 0.004; *OR* = 2.397, *p* = 0.003 respectively) (Table 3), indicating that these three significant polymorphisms could be risk factors of SCA.

**TABLE 3:** Alleles and genotypes frequency of rs20551, rs132788, and rs1805388 in all RPL involved exploratory and confirmatory sample (integrated).

Locus	SCA RPL (*n*= 54)	No SCA RPL (*n* = 88)	Control (*n* = 84)	SCA RPL vs. control		SCA RPL vs. no SCA RPL		No SCA RPL vs. control	
				*OR (95%CI)*	*p*	*OR (95%CI)*	*p*	*OR (95%CI)*	*p*
rs20551 (The East Asian frequency of allele G is 0.06311 in gnomAD v2.1.1 and 0.054 in 1000 Genomes Project Phase 3)									
A	92 (85.2)	167 (94.9)	164 (97.6)	0.140 (0.046-0.432)	0.000	0.310 (0.132-0.729)	0.005	0.453 (0.137-1.499)	0.259
**G**	16 (14.8)	9 (5.1)	4 (2.4)	**7.130 (2.315-21.96)**	**0.000**	**3.227 (1.372-7.591)**	**0.005**	2.210 (0.667-7.317)	0.259
AA	38 (70.4)	79 (89.8)	80 (95.2)	0.119 (0.037-0.379)	0.000	0.271 (0.110-0.668)	0.003	0.439 (0.130-1.484)	0.250
**AG**	16 (29.6)	9 (10.2)	4 (4.8)	**8.421 (2.635-26.91)**	**0.000**	**3.696 (1.497-9.124)**	**0.003**	2.278 (0.674-7.703)	0.250
GG	0 (0.0)	0(0.0)	0 (0.0)	—	—	—	—	—	—
rs132788 (The East Asian frequency of allele T is 0.2489 in gnomAD v2.1.1 and 0.233 in 1000 Genomes Project Phase 3)									
G	62 (57.4)	128 (72.7)	125 (74.4)	0.464(0.277-0.776)	0.004	0.505 (0.305-0.838)	0.008	0.917 (0.568-1.482)	0.807
T	46 (42.6)	48 (27.3)	43 (25.6)	2.157 (1.288-3.611)	0.004	1.978 (1.193-3.280)	0.008	1.090 (0.675-1.761)	0.807
GG	16 (29.6)	47 (53.4 )	52 (61.9)	0.259 (0.125-0.538)	0.000	0.367 (0.179-0.754)	0.008	0.705 (0.384-1.295)	0.283
GT	30 (55.6)	34 (38.6)	21 (25.0)	3.750 (1.808-7.777)	0.001	1.985 (0.999-3.947)	0.049	1.899 (0.982-3.634)	0.072
TT	8 (14.8)	7 (8.0)	11 (13.1)	1.154 (0.432-3.083)	0.804	2.012 (0.685-5.908)	0.197	0.574 (0.211-1.557)	0.324
rs1805388 (The East Asian frequency of allele A is 0.2288 in gnomAD v2.1.1 and 0.210 in 1000 Genomes Project Phase 3)									
G	73 (67.6)	130 (73.9)	140 (83.3)	0.417 (0.235-0.739)	0.003	0.565 (0.334-0.957)	0.036	0.738 (0.437-1.247)	0.280
A	35 (32.4)	46 (26.1)	28 (16.7)	2.397 (1.353-4.247)	0.003	1.769 (1.045-2.997)	0.036	1.355 (0.802-2.290)	0.280
GG	22 (40.7)	50 (56.8)	56 (66.7)	0.344 (0.169-0.697)	0.005	0.523 (0.263-1.039)	0.084	0.658 (0.354-1.222)	0.221
GA	29 (53.7)	30 (34.1)	28 (33.3)	2.320 (1.151-4.678)	0.022	2.243 (1.121-4.485)	0.024	1.034(0.550-1.947)	1.000
AA	3 (5.6)	8 (9.1)	0 (0.0)	0.588 (0.149-2.321)	0.553	—	—	—	—

Note: Values are number (percent) unless specified otherwise. Values in bold indicate statistically significantly (*p* < 0.05). The omission of Odds ratios (ORs), 95% confidence intervals (CIs) and *p*-value in the table was intentional because the number of cases was zero. RPL Recurrent Pregnancy Loss; SCA structural chromosome abnormalities; *CI* = confidence interval; *OR* = odds ratio.

Genotype frequencies of the rs20551, rs132788, and rs1805388 also were analyzed, as shown in Table 3, the rs20551 (AG), rs132788 (GT) and rs1805388 (GA) odds ratios were significantly greater in SCA RPL vs. no SCA RPL (Table 3, *OR* 3.696, 95% CI, 1.497–9.124, *p* = 0.003; *OR* 1.985, 95% CI, 0.999–3.947, *p* = 0.049 and *OR* 2.243, CI, 1.121–4.485, *p* = 0.024 respectively). However, no significant differences were observed in the frequencies of any combination of genotypes between affected RPL individuals and controls (*p* > 0.05, Table 4).

**TABLE 4:** Combination genotypes frequency of rs20551, rs132788, and rs1805388 in all RPL involved exploratory and confirmatory sample (integrated).

Genotypes	SCA RPL (*n* = 54)	no SCA RPL (*n* = 88)	control (*n* = 84)	SCA RPL vs. control		SCA RPL vs. no SCA RPL		No SCA RPLvs. control	
				*OR (95%CI)*	*p*	*OR (95%CI)*	*p*	*OR (95%CI)*	*p*
rs20551/rs132788/rs1805388									
AG/GG/GG	6 (11.1)	2 (2.3)	1 (1.2)	7.443 (1.750-19.01)	0.010	2.652 (1.043-7.079)	0.027	3.952 (0.433-16.11)	0.588
AA/GT/GG	13 (24.1)	10 (11.4)	9 (10.7)	3.369 (1.966-9.710)	0.036	1.905 (1.388-6.083)	0.046	1.504 (0.690-3.279)	0.893
AA/GG/GA	12 (22.2)	9 (10.2)	8 (9.5)	2.113 (1.047-3.268)	0.039	2.182 (0.177-3.430)	0.050	1.619 (0.348-2.153)	0.877
AA/GG/GG	13 (24.1)	23 (26.1)	28 (33.3)	0.613 (0.047-1.268)	0.245	0.918 (0.338-1.633)	0.784	0.582 (0.077-0.930)	0.301
AG/GT/GG	3 (5.6)	5 (5.7)	0	—	—	0.998 (0.791-1.870)	0.964	—	—
AA/TT/GG	3 (5.6)	5 (5.7)	8 (9.5)	0.688 (0.388-1.634)	0.401	0.998 (0.791-1.870)	0.964	0.675 (0.601-1.162)	0.341
AG/TT/GG	1 (1.9)	2 (2.3)	1 (1.2)	1.538 (0.168-6.427)	0.751	0.828 (0.102-3.855)	0.865	1.654 (0.226-12.09)	0.588
AA/GT/GA	4 (7.4)	11 (12.5)	10 (11.9)	0.669 (0.266-4.710)	0.393	0.514 (0.190-2.279)	0.338	1.105 (0.338-5.083)	0.906
AG/GG/GA	3 (5.6)	8 (9.1)	7 (8.3)	0.603 (0.250-3.017)	0.539	0.652 (0.433-3.101)	0.444	1.012 (0.643-2.791)	0.860
AG/GT/GA	0	0	0	—	—	—	—	—	—
AA/TT/GA	2 (3.8)	4 (4.6)	3 (3.6)	1.008 (0.318-2.637)	0.964	0.922 (0.179-2.725)	0.808	1.475 (0.441-2.172)	0.747
AG/TT/GA	1 (1.9)	2 (2.3)	2 (2.4)	0.828 (0.131-2.428)	0.836	0.828 (0.102-3.855)	0.865	0.932 (0.257-4.543)	0.964
AA/GG/AA	2 (3.8)	2 (2.3)	0	—	—	1.748 (0.367-2.733)	0.617	—	—
AA/GT/AA	2 (3.8)	2 (2.3)	0	—	—	1.748 (0.367-2.733)	0.617	—	—
AG/GG/AA	1 (1.9)	1 (1.1)	0	—	—	1.752 (0.533-1.111)	0.726	—	—
AG/GT/AA	0	0	0	—	—	—	—	—	—
AA/TT/AA	1 (1.9)	0	0	—	—	—	—	—	—
AG/TT/AA	0	0	0	—	—	—	—	—	—

Note: Values are number (percent) unless specified otherwise. The omission of Odds ratios (ORs), 95% confidence intervals (CIs) and *p*-value in the table was intentional because the number of cases was zero. RPL = Recurrent Pregnancy Loss; SCA = structural chromosome abnormalities; *CI* = confidence interval; *OR* = odds ratio.

### Frequencies of Translocations in No SCA Samples With Different Genotypes

**FIGURE 3:**
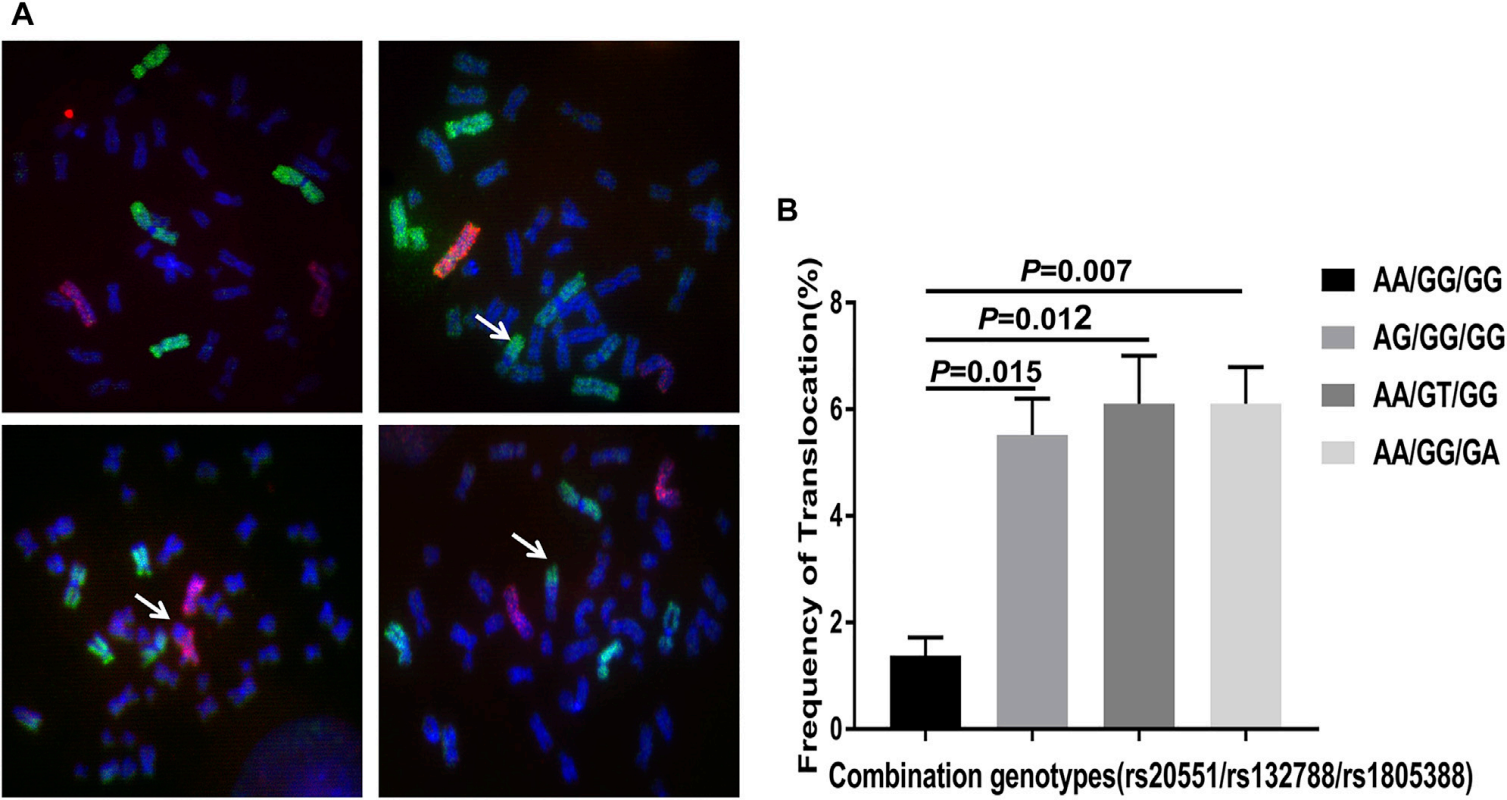
Fluorescence *in situ* hybridization (FISH) using chromosome 1 probe (green), chromosome 2 probe (red) and chromosome 4 probe (green) (Cytocell, Cambridge, United Kingdom). **(A)** Representative images of photomicrographs showing FISH painted human chromosome 1, 4 (green) and chromosome 2 (red) in metaphase lymphocytes. No translocation in the upper left image, the upper right showing translocation involving chromosome 1 (arrow), the bottom left showing translocation involving chromosome 2 (arrow), the bottom right showing translocation involving chromosome 4 (arrow). **(B)** Analysis of the frequency of radiation-induced chromosome translocations in no SCA patients with different genotypes. The statistical analysis chart showing the frequencies of radiation-induced translocations in patients with AG/GG/GG (rs20551), AA/GT/GG (rs132788) and AA/GG/GA (rs1805388) genotype were significantly higher than that in AA/GG/GG (wild type) (*p* = 0.015, *p* = 0.012, *p* = 0.007 respectively). The experiments were repeated three times. FISH: fluorescent *in situ* hybridization; SCA: structural chromosome abnormality.

To further confirm the association of significant SNPs (rs20551/rs132788/rs1805388) with SCA, FISH was used to detect the radiation-induced chromosome translocations (the most common SCA) in different genotype peripheral blood lymphocytes (PBLs) from no SCA RPL after 2Gy X-rays. The result demonstrates the frequencies of radiation-induced chromosome translocations in AG/GG/GG, AA/GT/GG and AA/GG/GA PBLs were significantly higher than that in AA/GG/GG (wild type) PBLs (Figure 3, *p* = 0.015, *p* = 0.012, *p* = 0.007 respectively).

## Discussion

In the present study, the potential association of *EP300*, *XRCC6*, *LIG4*, *XRCC4*, *PRKDC*, and *DCLRE1C* genes polymorphisms with structural chromosome abnormality (SCA) has been investigated by targeted whole-exome sequencing for the first time. *EP300* rs20551, *XRCC6* rs132788, and *LIG4* rs1805388 frequencies were statistically significantly different between RPL with SCA and RPL without SCA. Moreover, no SCA peripheral blood lymphocytes (PBLs) with rs20551, rs132788, or rs1805388 locus were more prone to translocation after radiation. These findings provide evidence that DNA repair related genes polymorphisms could be an important contributor to the risk of SCA.

From few studies on the association of gene polymorphisms with SCA, one found a significant decrease in the distribution of T allele in *MTHFR* 677C > T polymorphisms among patients with chromosomal abnormalities (Sinthuwiwat et al., 2012). The rs231775 and rs3087243 of *CTLA4*, as well as rs2232365 and rs2232368 of *Foxp3*, all appeared to have chromosomal abnormalities (Fan et al., 2018). Before the present study, no gene polymorphism within *EP300*, *XRCC6* and *LIG4* genes was reported associated with SCA.

*EP300* functions as histone acetyltransferase that regulates transcription via chromatin remodeling (Lundblad et al., 1995), plays a critical role in SCA. Histone acetyltransferase modification is considered to be an important factor in the formation of chromosomal translocation (Burgess, 2015). The acetylation of histone enrolls chromatin remodeling complexes to the nearby double-strand breaks (DSBs) sites, promoting the process of DNA damage repair (DDR) (Lee et al., 2010). It is known that DDR is considered to be the initiating molecular event in the formation of chromosome translocation (Nambiar and Raghavan, 2011). The rs20551 is a non-synonymous single nucleotide variant in *EP300* locates on chromosome 22, with the change of c.2989A > G, resulting in the substitution of valine for isoleucine at codon 997 close to the Bromodomain (Li et al., 2017). It is known that the Bromodomain is a protein domain that recognizes acetylated lysine residues, and the recognition could be affected when some changes occur nearby. In our study, the frequency of G allele in rs20551 was significantly higher in SCA group than no SCA group, indicating that G allele in rs20551 could be a risk factor to SCA. The tentative explanation is that the acetylation of EP300 may be affected when the *EP300* rs20551 is present, and the normal DNA repair pathways EP300 involved may also be affected as a consequence.

*XRCC6* encodes the Ku 70 protein, which is crucial to repairing DSBs in identifying broken ends of DNA. In the process of DNA damage repair (DDR), Ku heterodimer composed of Ku 70 and Ku 80 binds to the broken DNA as the first molecule (Chanut et al., 2016), and a recruitment platform for subsequent repair enzymes is established (Williams et al., 2014). The basic steps of DDR have been biochemically defined to require DSBs detection by the Ku heterodimer, which functions in combination with XRCC4 and XLF (Williams et al., 2014). The rs132788 is a synonymous variant in *XRCC6* with the change of c.1629G > T. Although the encoded amino acids not be changed (Gly > Gly), rate of protein synthesis could be influenced as the codon changes (Koutmou et al., 2015). A review and meta-analysis on risk factors for breast cancer showed that rs132788 (G > T) might be protective (Zhou et al., 2012), while another study suggested that the rs132788 polymorphism may be a susceptibility factor for radiation-induced oral mucositis in Chinese nasopharyngeal carcinoma patients (Ren et al., 2014). In our study, the frequency of the T allele in the *XRCC6* rs132788 locus was significantly higher in the SCA affected individuals, clearly suggesting that rs132788 could be a susceptibility factor to SCA, filling in the gap of clinical significance reported in ClinVar database.

DNA LIG4 is essential for V(D)J recombination and DNA double-strand breaks (DSBs) repair through non-homologous end joining (NHEJ) (Grawunder et al., 1998; Zhao et al., 2020). Defects in LIG4 could lead to pronounced radio-sensitivity and confer a predisposition to leukemia (Riballo et al., 1999). Rs1805388 in LIG4 was also reported associated with increased radio-resistance (Mumbrekar et al., 2016). One study claimed the rs1805388 gene polymorphism is not a risk factor of cancer (Xie et al., 2014), while another study reported rs1805388 was associated with an increased glioma risk among smokers (Zhao et al., 2013). Additionally, LIG4 rs1805388 was also associated with susceptibility to male infertility (Ji et al., 2013). Our results showed the rs1805388 was strongly associated with SCA.

Although the SCA cases we used were derived from recurrent pregnancy loss (RPL), the three significant polymorphisms we found were not associated with RPL. When the no SCA.

RPL was compared to normal control, no significant polymorphism was found. The evidence is more robust that rs20551, rs132788 and rs1805388 are associated with the risk of SCA rather than RPL.

As one of the most important types of SCA, translocation is often assumed to form because of the joining of DSBs that arise at different sites on non-homologous chromosomes (Bunting and Nussenzweig, 2013). One study suggests that Ku70 can increase DSB rejoining and translocation levels in *LIG4*-deficient G1-arrested progenitor B cells (Liang et al., 2021). Translocations were also increased in a reporter system in mouse embryonic stem cells when *XRCC4*–XLF was inactivated (Simsek and Jasin, 2010). Our results also show that polymorphisms within *EP300*, *XRCC6* (Ku70), and *LIG4* might affect the risk of translocation.

Despite sufficient powerful mastery and analysis, one of the limitations of our study might be the relatively small sample size, which does not allow definite conclusion, especially for the analysis of the interaction between combined genotypes. Another limitation is only six genes in RPL women have been analyzed. Future studies of the other SCA cases are needed. Nevertheless, this study has several strengths including the use of human peripheral blood samples for analysis, case-control and inclusion of typical clinical affected individuals with SCA. Significantly higher frequencies of *EP300* rs20551 (A/G), *XRCC6* rs132788 (G/T) and *LIG4* rs1805388 (G/A) were found in SCA group.

In conclusion, our study improved the understanding of genetic polymorphisms within the *EP300*, *XRCC6*, *LIG4*, *XRCC4*, *PRKDC*, and *DCLRE1C* genes with structure chromosomal abnormalities (SCA). *EP300* rs20551, *XRCC6* rs132788 and *LIG4* rs1805388 might be associated with the risk of SCA. This all could be useful in guiding future research into molecular mechanisms of SCA and uncovering the partial pathogenesis of human diseases caused by SCA. Moreover, these significant polymorphisms might also be valuable diagnostic markers and potential therapy targets for the affected RPL individuals with SCA.

## Data Availability

The data that support the findings of this study are available from the corresponding author upon reasonable request.
