# Supplementary material for: Polymorphisms Within DNA Double-Strand Breaks Repair-Related Genes Contribute to Structural Chromosome Abnormality in Recurrent Pregnancy Loss
Source: Front Genet. 2021 Dec 23;12:787718. doi: 10.3389/fgene.2021.787718 (PMC8733605; doi:10.3389/fgene.2021.787718)
Supplement: Supplementary file 1 [file DataSheet1.ZIP › Supplementary data/Supplemental Table S3.docx]

**TABLE S3**

**Karyotype results of 142 clinical RPL Patients**

**Patient number Carrier status**

***Reciprocal translocation***

1 46,XX,t(11;14)(q21;q32)

2 46,XX,t(15;16)(q25;q21)

3 46,XX,t(2;10)(q34;p12)

4 46,XX,t(7;14)(q22;q13)

5 46,XX,t(2;4)(q31;q24),t(9;11)(p10;p10)

6 46,XX,t(4;12)(q31;q24)

7 46,XX,t(2;12)(q37;q13)

8 46,XX,t(12;18)(q21;q12)

9 46,XX,t(1;4)(p34;p16)

10 46,XX,t(11;21)(q13;q22)

11 46,XX,t(1;15)(p36.2;q25)

12 46,XX,t(1;5)(q42;q22)

13 46,XX,t(7;15)(q22;q24)

14 46,XX,t(2;10)(p13;p11)

15 46,XX,t(10;18)(p11.2;q23)

16 46,XX,t(5;15)(q15;q26.1)

17 46,XX,t(13;20)(q32;p13)

18 46,XX,t(9;10)(p24;p11)

19 46,XX,t(2;12)(q37;q24)

20 46,XX,t(4;15)(q21;q11.2)

21 46,XX, t(9;12)(q22;q13)

22 46,XX,t(1;20)(p13;p11.2)

23 46,XX,t(2;12)(q37;q13)

24 46,XX,t(11;22)(q23;q11.2)

25 46,XX,t(3;18)(p13;q21.1)

26 46,XX,t(3;14)(p23;q21)

27 46,XX,t(2;4)(q33;q31)

28 46,XX,t(5;7)(q31;q36)

29 46,XX,t(8;11)(q22;q23)

30 46,XX,t(4;11)(q33;q21)

31 46,XX,t(1;15)(q32.3;q26.1)

32 46,XX,t(7;20)(p10;p10)

***Robertsonian translocation***

33 45,XX,der(13;14)(q10;q10)

34 45,XX,der(14;21)(q10;q10)

35 45,XX,der(13;14)(q10;q10)

36 45,XX,der(13;21)(q10;q10)

37 45,XX,der(13;14)(q10;q10)

38 45,XX,der(13;15)(q10;q10)

39 45,XX,der(15;22)(q10;q10)

40 45,XX,der(13;14)(q10;q10)

41 45,XX,der(13;14)(q10;q10)

42 45,XX,der(13;14)(q10;q10)

43 45,XX,der(13;14)(q10;q10)

44 45,XX,der(13;14)(q10;q10)

45 45,XX,der(13;14)(q10;q10)

46 45,XX,der(13;22)(q10;q10)

***Inversion***

47 46,XX,inv(9)(p11q13)

48 46,XX,inv(9)(p11q13)

49 46,XX,inv(9)(p11q13)

50 46,XX,inv(9)(p11q13)

51 46,XX,inv(9)(p11q13)

52 46,XX,inv(9)(p11q13)

53 46,XX,inv(9)(q22q34)

54 46,XX,inv(8)(p12q22)

***Normal karyotype***

55-142 46,XX

*Note:* RPL = recurrent pregnancy loss.
