# Supplementary figures and images for: Polymorphisms Within DNA Double-Strand Breaks Repair-Related Genes Contribute to Structural Chromosome Abnormality in Recurrent Pregnancy Loss
Source: Front Genet. 2021 Dec 23;12:787718. doi: 10.3389/fgene.2021.787718 (PMC8733605; doi:10.3389/fgene.2021.787718)

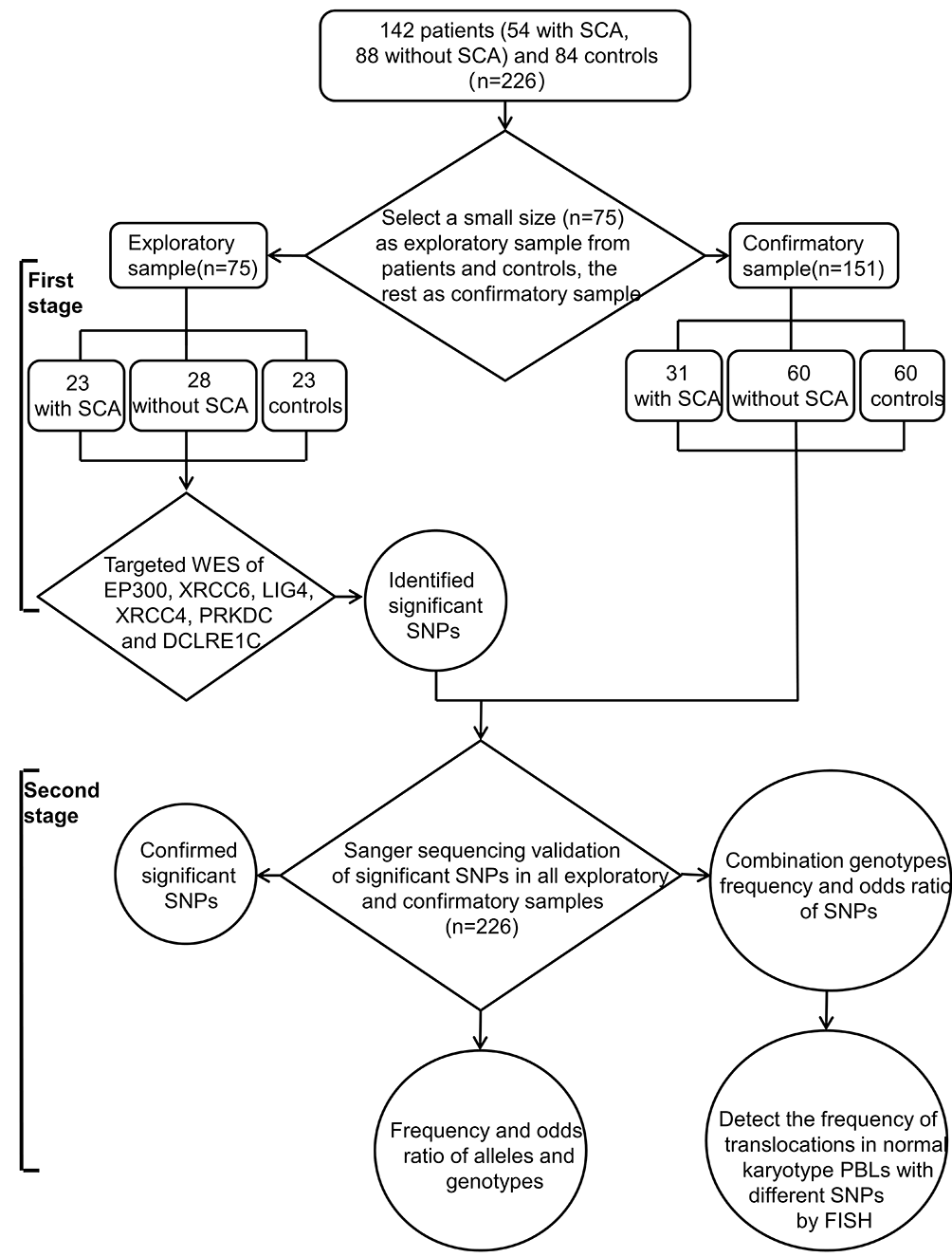

Supplement: Supplementary file 2 [file DataSheet2.ZIP › Figures/Figure 1.tif]

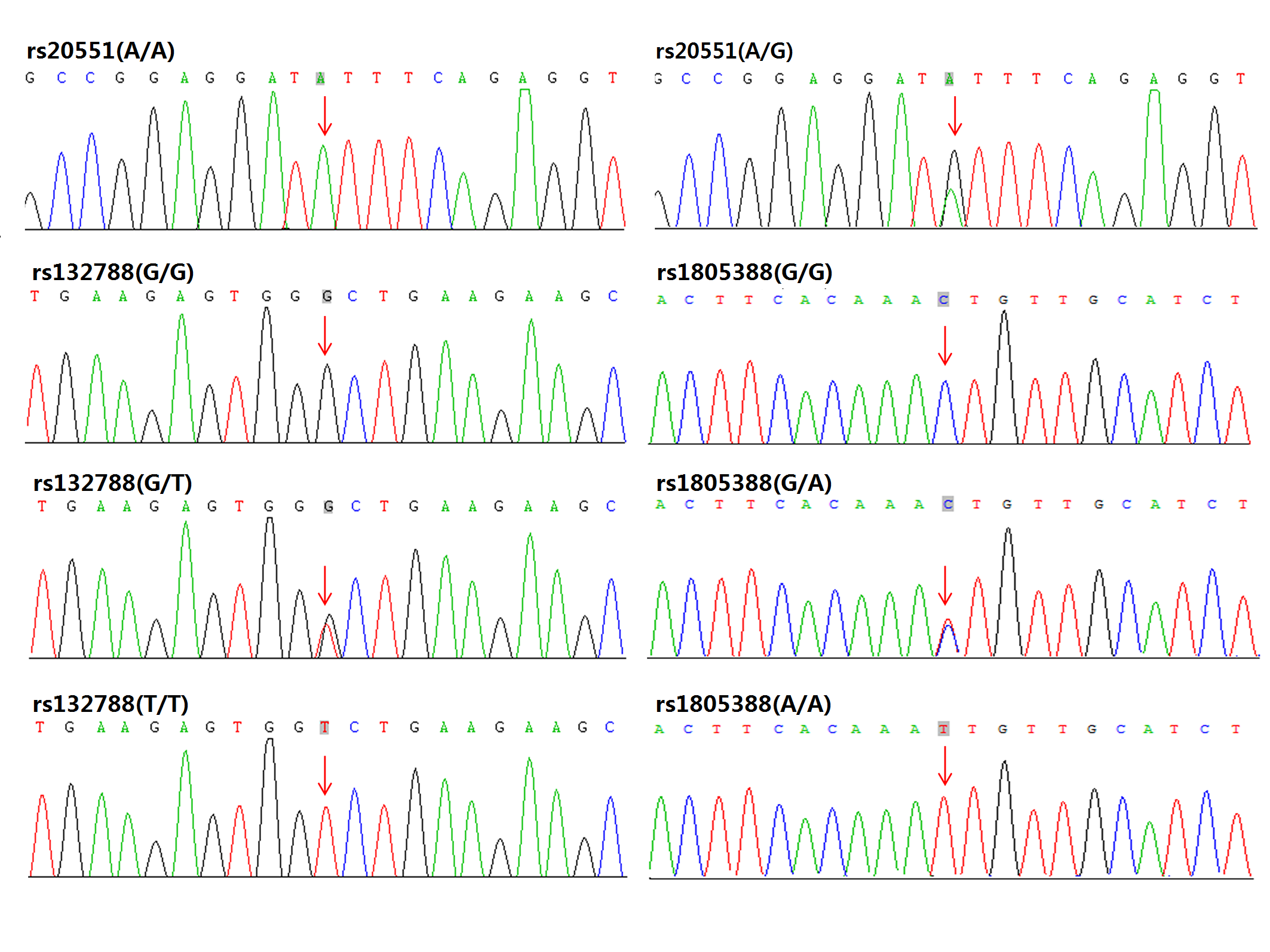

Supplement: Supplementary file 2 [file DataSheet2.ZIP › Figures/Figure 2.tif]

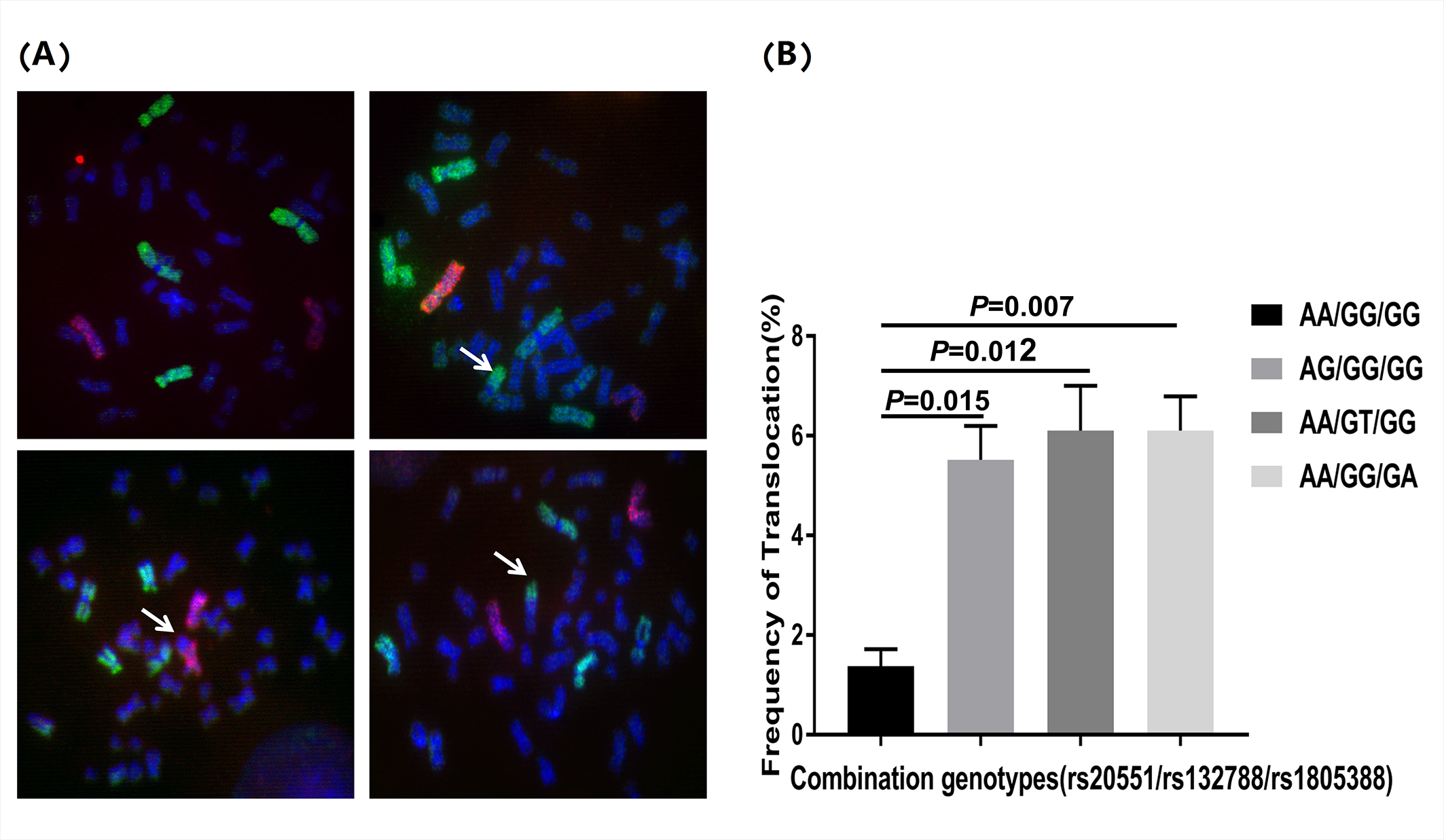

Supplement: Supplementary file 2 [file DataSheet2.ZIP › Figures/Figure 3.tif]
