# Supplementary material for: Polymorphisms Within DNA Double-Strand Breaks Repair-Related Genes Contribute to Structural Chromosome Abnormality in Recurrent Pregnancy Loss
Source: Front Genet. 2021 Dec 23;12:787718. doi: 10.3389/fgene.2021.787718 (PMC8733605; doi:10.3389/fgene.2021.787718)
Supplement: Supplementary file 1 [file DataSheet1.ZIP › Supplementary data/Supplemental Table S2.doc]

**TABLE S2**

**Primers used in Sanger sequencing**

| **SNPs** | **Forward** | **Reverse** |
| --- | --- | --- |
| rs20551 | 5’- CCAGCTGTAAGCATTGAAGGA -3’ | 5’- TGAACACCCTTTCTCATGCAA -3’ |
| rs132788 | 5’- GCCACTTGTATGTAGCATTTCC -3’ | 5’- GTCGGGTAGACTCTTCCTAGCTC -3’ |
| rs1805388 | 5' -GCAAAACAGTTATTAAATGTAGAGG-3' | 5' –AGAGTCTGTGACATCTTTGTGGTTC-3' |
| rs188035979 | 5’- TCTAATCCACAACCCATCCCT-3’ | 5’- TGCTGTCGTCTCAAGATGTCTC-3’ |
| rs17224367 | 5’- AAAGTGCTGGGATTACAGGTGT-3’ | 5’- TTACCTTCATGTTTTTCCAGAGC-3’ |
| rs20552 | 5’-ACCAACTTTATGAGGACAGCACA-3 | 5’-TGTCTAGAGTAACAGGGACCAAAG-3’ |
| rs17002307 | 5’-TGTCTAGAGTAACAGGACCAAAG-3’ | 5’-AAAACCAGACACTCACTCGATTG-3’ |
| rs55778204 | 5’-TTTGTCCCTAAGGAGTTGTTCG-3’ | 5’-AAAGCCAGGTGACATTCAGAAC-3’ |
| rs20554 | 5’-TTGCATCCGCAGAGCCTC-3’ | 5’-TCCCATCAACTCTCCATTCAA-3’ |
| rs78045947 | 5’-GCAGAGCTCACCTGAGGAAC-3’ | 5’-GAGGGCCCTGTGGTTATCA-3’ |
| rs188035979 | 5’-CCCCGCTCCTCTCAGTAGTCT-3’ | 5’-GCCAACACCCCCAAGTACA-3’ |
| rs146242251 | 5’-GCCCGTTTGTCCTCTAATT-3’ | 5’-AATTAGAGGACAAACGGGC-3’ |
| rs56170584 | 5’-CCAAACCTTCGGACGGAAA-3’ | 5’-TTTCCGTCCGAAGGTTTGG-3’ |
| rs55778204 | 5’-TCAGTGGTTCGTAGGGCTT-3’ | 5’-AAGCCCTACGAACCACTGA-3’ |
| rs12768894 | 5’- TCTAATCCACAACCCATCCCT-3’ | 5’- TGCTGTCGTCTCAAGATGTCTC-3’ |
| rs1805389 | 5’- AAAGTGCTGGGATTACAGGTGT-3’ | 5’- TTACCTTCATGTTTTTCCAGAGC-3’ |
| rs139713386 | 5’-ACCAACTTTATGAGGACAGCACA-3 | 5’-TGTCTAGAGTAACAGGGACCAAAG-3’ |
| rs2232641 | 5’-TGTCTAGAGTAACAGGACCAAAG-3’ | 5’-AAAACCAGACACTCACTCGATTG-3’ |
| rs11411516 | 5’-TTTGTCCCTAAGGAGTTGTTCG-3’ | 5’-AAAGCCAGGTGACATTCAGAAC-3’ |
| rs55769154 | 5’-TTGCATCCGCAGAGCCTC-3’ | 5’-TCCCATCAACTCTCCATTCAA-3’ |
| rs55793951 | 5’-GCAGAGCTCACCTGAGGAAC-3’ | 5’-GAGGGCCCTGTGGTTATCA-3’ |
| rs750714859 | 5’-CCCCGCTCCTCTCAGTAGTCT-3’ | 5’-GCCAACACCCCCAAGTACA-3’ |
| rs756127946 | 5’-GCCCGTTTGTCCTCTAATT-3’ | 5’-AATTAGAGGACAAACGGGC-3’ |
| rs77033659 | 5’-CCAAACCTTCGGACGGAAA-3’ | 5’-TTTCCGTCCGAAGGTTTGG-3’ |
| rs749856389 | 5’-TCAGTGGTTCGTAGGGCTT-3’ | 5’-AAGCCCTACGAACCACTGA-3’ |
| rs187813872 | 5’- TCTAATCCACAACCCATCCCT-3’ | 5’- TGCTGTCGTCTCAAGATGTCTC-3’ |
| rs369274149 | 5’- AAAGTGCTGGGATTACAGGTGT-3’ | 5’- TTACCTTCATGTTTTTCCAGAGC-3’ |
| rs547031184 | 5’-ACCAACTTTATGAGGACAGCACA-3 | 5’-TGTCTAGAGTAACAGGGACCAAAG-3’ |
| rs183622528 | 5’-TGTCTAGAGTAACAGGACCAAAG-3’ | 5’-AAAACCAGACACTCACTCGATTG-3’ |
| rs1805389 | 5’-TTTGTCCCTAAGGAGTTGTTCG-3’ | 5’-AAAGCCAGGTGACATTCAGAAC-3’ |
| rs139713386 | 5’-TTGCATCCGCAGAGCCTC-3’ | 5’-TCCCATCAACTCTCCATTCAA-3’ |
| rs2232641 | 5’-GCAGAGCTCACCTGAGGAAC-3’ | 5’-GAGGGCCCTGTGGTTATCA-3’ |
| rs77033659 | 5’-ACCAACTTTATGAGGACAGCACA-3 | 5’-TGTCTAGAGTAACAGGGACCAAAG-3’ |
| rs749856389 | 5’-TGTCTAGAGTAACAGGACCAAAG-3’ | 5’-AAAACCAGACACTCACTCGATTG-3’ |
| rs187813872 | 5’-TTTGTCCCTAAGGAGTTGTTCG-3’ | 5’-AAAGCCAGGTGACATTCAGAAC-3’ |
| rs369274149 | 5’-TTGCATCCGCAGAGCCTC-3’ | 5’-TCCCATCAACTCTCCATTCAA-3’ |
| rs547031184 | 5’-GCAGAGCTCACCTGAGGAAC-3’ | 5’-GAGGGCCCTGTGGTTATCA-3’ |
